# Association of Morphological Varieties of Soft Palate and Airway Measurement Among Riyadh Population: A Retrospective Cephalometric Analysis

**DOI:** 10.1155/ijod/9882321

**Published:** 2025-06-19

**Authors:** Amara Swapna Lingam, Mohammad Albelaihi, Saad Alshamrani, Nawaf AlHababi, Abdullah Rayash, Lama Al Qahtani, Rawa Kamal Abdelrahim, Tahseen Ali Khan, Shaimaa F. K. Habib, Pradeep Koppolu

**Affiliations:** ^1^UWA Dental School, The University of Western Australia, Perth, Australia; ^2^College of Dentistry, Dar al Uloom University, Riyadh, Saudi Arabia

**Keywords:** airway, lateral cephalogram, soft palate

## Abstract

**Background:** It is anticipated that the size and morphology of the airways will shift because of the development of the maxillofacial bone and the soft tissues. The soft palate plays a major role in various functions and may have an impact over the changes in the airway.

**Aim:** To evaluate the association of morphological varieties of soft palate and airway measurement among three different age-groups of Riyadh population.

**Methodology:** Patients who were obese, had mild-to-moderate breathing issue, and young patients seeking orthodontic treatment were advised to get lateral cephalogram. Further, they were divided into three age-groups and the upper airway diameter (UAD), mandibular plane hyoid distance (MN-HY) was measured. The data collected were subjected to statistical analysis.

**Result:** This study involved 831 patients, among which, 31–50-year-olds had greater mean UAD (12.54 ± 3.74), whereas 50+ had higher MN-HY (13.23 ± 6.22). S-shaped soft palate had greater mean UAD (19.63 ± 8.14), but straight line-shaped had higher MN-HY (13.94 ± 5.8). People between the ages of 10 and 30 (100%) had an undefined soft palate shape, while those between 31 and 50 years (38.2%) had a crooked one and those over 50 (34.1%) had a straight one (*p* < 0.05). Multivariate linear regression demonstrates that the MN-HY ( = 1.607; *p* < 0.001) is substantially associated with older age-groups (*p* < 0.001). The majority of straight-line soft palates were associated with the majority of normal range UAD and MN-HY (*p* < 0.01) values.

**Conclusion:** A significant association found between the changes in soft palate and the size of the airway. Middle aged patients showed higher UAD, while the older age group showed higher MN-HY.

## 1. Introduction

The term “soft palate” refers to the fibromuscular portion of the posterior region of the palate that is linked to the hard palate which is formed by the fusion of three different component [1]. These components are the primitive palate, which is created by the front nasal process, as well as the two palatal processes. Following this stage, the mesoderm in the palate goes through a process called intramembranous ossification, which results in the formation of the hard palate [2]. However, the ossification does not continue into the most posterior part, therefore, the soft palate continues to be present after this process [3].

The soft palate is involved in the majority of oral activities, such as velopharyngeal closure, it contributes to the processes of sucking, swallowing, and pronouncing. During swallowing, the soft palate separates the mouth from the oropharynx, which allows respiration to remain undisturbed, allows for the quality of the voice to be altered, and allows for the correct pronunciation of consonants [2].

Lateral cephalogram is a well-known way to look at the different shapes of the soft mouth, which in normal people comes in six different forms with different lengths for each. You et al. [4] evaluated the velum's appearance on lateral cephalograms and divided them into six morphological types: Type 1 is leaf-shaped, Type 2 is rat-tail-shaped, Type 3 is butt-like, Type 4 is a straight line, Type 5 is S-shaped, and Type 6 is crook-shaped. According to Pepin et al. [5], awake patients with a “hooked or S-shaped” soft palate had a high probability of developing obstructive sleep apnea (OSA) syndrome.

With the development of the maxillofacial bone and soft tissues, changes in airway size and morphology are anticipated [6]. Studies on the Riyadh population, however, are limited. Thus, the present study was undertaken to see if there is a link between changes in the shape of the soft palate and the size of the airway in a sample of people from three different age groups: 10–30, 31–50, and 51–70 years.

## 2. Methodology

This is a retrospective study conducted at the University Hospital, Dental Clinics, Riyadh, Saudi Arabia. The software used to retrieve and analyze the images is Sidexis software available in the clinics.

The institution's ethical committee reviewed and approved the study with an ethical number 014-03-2023. The sample size of 831 was estimated using a single proportion method with an expected proportion of 0.649, a relative precision of 5%, and a 95% confidence level. Patients who attended the dental clinics were selected for the study by sampling using purposive sampling technique. All the patients who were obese had mild/moderate complaints of sleep or breathing issues and who had come for orthodontic consultation were included in the study. They were advised cephalometric radiographs in the clinic as a regular protocol and all the retrospective data of images which had no errors were considered in the current study. Any patient with eight or more missing teeth, patients with previous history of cleft palate surgeries, or previous orthognathic/orthodontic treatment were excluded from the study. Any radiographs that had error in positioning or machine related were also excluded from the study. Following the selection, the patients were divided into three groups depending no their age: 10–30, 31–50, and 51–70 years.

Digital lateral cephalograms were recorded using the orthopantomographic (Sirona Company) equipment. A 84 kV, 13 mA for 9.4 s of tube potential adjustment was made to enhance the contrast of both hard and soft tissues.

The distance between the closest point on the outer pharyngeal wall and the point on the anterior half of the soft palate was used to calculate the upper airway diameter (UAD) [7]. Drawing a tangent to the mandibular plane and measuring the distance to the hyoid bone from that tangent were used to determine the MP-H. In order to eliminate bias brought on by interobserver and intraobserver variability, both measurements were taken by two observers at two different times separated by 10 days interval.

## 3. Statistical Methods

The collected data were analyzed using SPSS version 28. Descriptive statistics like mean, standard deviation, frequency, and percentages were calculated. Chi-square analysis was performed to measure the association between age groups with shape of soft palate and between different soft palate shapes with UAD and mandibular plane hyoid distance (MN-HY) range. Multivariate linear regression analysis was performed to measure the association between age groups and UAD and MN-HY levels.

## 4. Results

A total of 831patients, both males and females were enrolled in this study.  Table 1 displays the distribution of age groups and shape of soft palate among UAD and MN-HY of study population. Age group of 31–50 years displayed higher UAD mean (12.54 ± 3.74), whereas above 50 years showed higher MN-HY levels (13.23 ± 6.22). Similarly, S-shaped soft palate displayed higher mean UAD (19.63 ± 8.14), whereas straight line shaped soft palate showed higher MN-HY levels (13.94 ± 5.8; Table 1).

**Figure 1:**
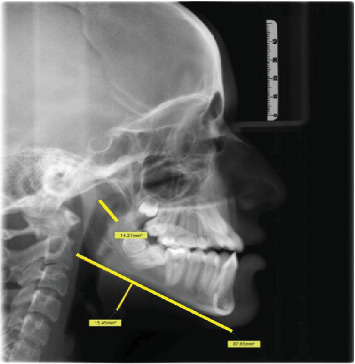
Rat-tail shape with the UAD and MAN-HY measurements.

**Figure 2:**
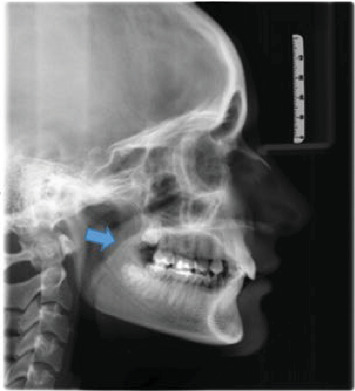
Straight line shape.

**Figure 3:**
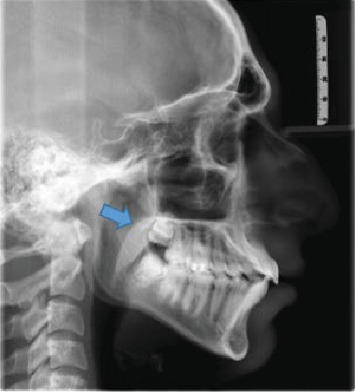
Leaf shape.

**Figure 4:**
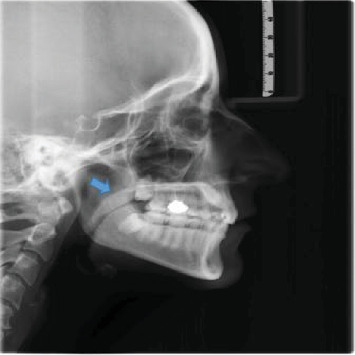
Crooke shape.

**Figure 5:**
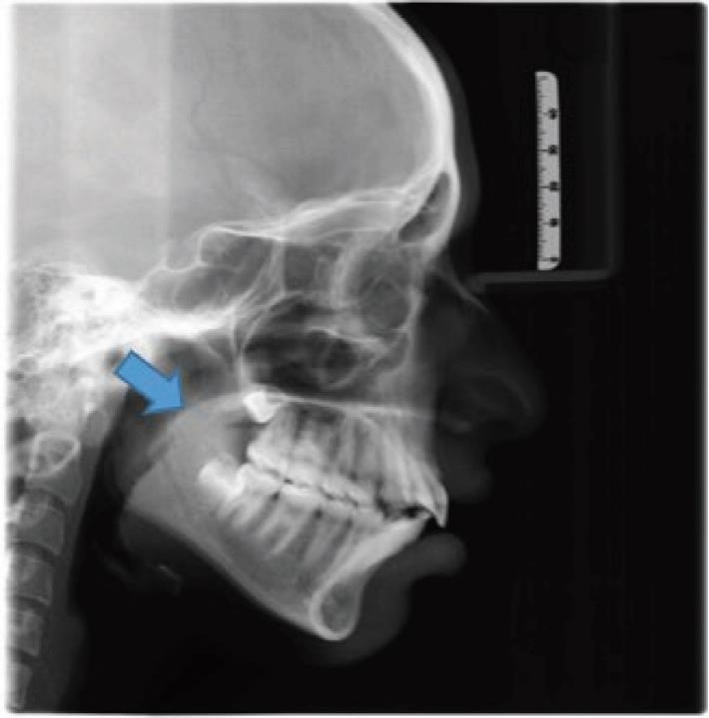
Butt-like shape.

**Figure 6:**
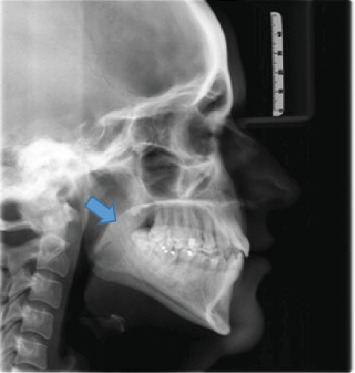
S-shape.

Figure 1 displays the measurement of UAD and MN-HY with the shape of soft palate identified in the image. Figures 2–6 depict the presentation of different shapes of soft palate among the study population.

A Chi-square test displays a statistically significant relationship between age groups and shape of soft palate (*χ*^2^ (12) = 84.52, *p* < 0.001). This shows that the majority of people between the ages of 10 and 30 (100%) are associated with an undefined soft palate shape, while people between the ages of 31 and 50 (38.2%) exhibited a crooked type of soft palate shape, while the majority of people with a straight palate shape (34.1%) were people over the age of 50 (Table 2).

Multivariate linear regression displays the MN-HY (*β* = 1.607; *p*  < 0.001) was significantly associated with higher age groups. The results clearly direct the positive effect of higher age group with MN-HY. Moreover, the *R*^2^ = 0.038 depicts that the model explains 3.8% of variance in MN-HY (Table 3).

Chi-square analysis revealed a significant association between different soft palate shapes and UAD and MN-HY range. The majority of straight line soft palates were associated with the majority of normal range of UAD (*χ*^2^ value = 13.96; *p*=0.03) and normal range of MN-HY (*χ*^2^ value = 64.5; *p*  < 0.001; Table 4).

## 5. Discussion

The current study was undertaken to find if there exists any association between the changes in the shape of the soft palate and the size of the airway in three different age groups. As per the available literature, this is one of the first studies conducted among Riyadh population.

For orthodontic purposes, lateral cephalograms are often utilized radiographs. According to Maltais et al. [8], using cephalometric analysis to evaluate the upper airway anatomy is beneficial because it is less complicated than other techniques for determining airway patency. Pirilä-Parkkinen et al. [9] suggested that lateral cephalograms are a legitimate tool for determining the dimensions of the nasopharynx and retropalatal area served as additional confirmation. Hence, lateral cephalograms were used in the present study.

In the present study, it was seen that age group of 31–50 years displayed higher UAD mean, whereas above 50 years showed higher MN-HY levels. According to Goncalves et al. [10], UAD increases with age, but lower airway growth varies between ages 6 and 18. S-shaped soft palate displayed higher mean UAD, whereas straight line shaped soft palate showed higher MN-HY levels. A statistically significant relationship between age groups and shape of soft palate was seen, which shows that the majority of people between the ages of 10 and 30 are associated with an undefined soft palate shape, while the majority of people with straight palate shape were people over the age of 50. Although not significant, similar results were obtained by Maiti et al. [11], where the length and the maximum width of the soft palate was found to be more in age groups of 31–40 years. According to Abramson et al. [12], younger patients' airways were shorter, more narrow, and smaller than those of older patients. In general, the cross section of younger patients' airways was less elliptical, more homogeneous, and more compact.

Multivariate linear regression shows that the MN-HY was substantially related with older age groups in the current investigation. The findings showed that as the age increased MN-HY increased too. Similar results were obtained by Lavanya et al. [13], where they saw significantly higher MN-HY values in the older age group. Different soft palate shapes were found to significantly correlate with the UAD and MN-HY range. The majority of normal range of UAD and normal range of MN-HY correlated with the majority of straight line soft palates. The pharynx is made up of a tube made of soft tissue that is supported by bone structure. The size and shape of the airways are determined in part by the tension and size of the soft tissues, as well as the position of the bone attachments. Consequently, it is anticipated that changes in airway size and morphology will occur concurrently with the development of the maxillofacial bone and soft tissues [6, 14]. This could be the reason for the results of the current study.

The findings of this study have important clinical implications for the Riyadh population, particularly in assessing airway dimensions and soft palate morphology. The association between higher MN-HY levels in older individuals and variations in soft palate shapes highlights the need for age-specific evaluations in airway management. Given the rising prevalence of OSA in Riyadh, these insights can aid in early screening and personalized treatment planning. Similar studies have shown the correlation between soft palate morphology and airway dimensions, reinforcing the importance of anatomical assessments in predicting airway-related disorders [1, 3, 4]. These findings have broader applicability beyond the Riyadh region, as the observed associations between age, soft palate morphology, and airway dimensions provide valuable insights for early screening and management of airway-related disorders in diverse populations. Future research should focus on longitudinal studies to assess the progression of airway changes over time and their clinical implications. Additionally, studies with larger and more diverse samples, including different ethnic groups and geographic regions, are needed to validate these findings and enhance their generalizability.

Soft palate morphology can serve as an important screening tool for respiratory conditions, particularly OSA, by identifying anatomical variations that may contribute to airway obstruction. Studies have demonstrated that specific soft palate shapes, such as the hooked or S-shaped palate, are associated with altered airway dimensions, impacting airflow and increasing susceptibility to OSA [1, 5]. In dentistry, assessing soft palate structure is crucial for managing conditions like malocclusion and prosthetic appliance fittings, as variations in soft tissue anatomy can influence oral function and airway stability [3]. Integrating soft palate evaluation into routine clinical assessments can enhance early detection of airway-related disorders and guide personalized treatment planning in both respiratory and dental care.

## 6. Limitation

In the current study, standardization of calculation of body mass index (BMI) was not possible. If there were significant differences in BMIs across groups, this could have had an impact on airway changes in this investigation because airway size and shape may be affected by BMI [15]. In the current study, we analyzed retrospectively the changes in soft palate changes with different age groups. The relatively small sample size and the single-center design of the study prevent the generalization of the findings. To further understand the morphology of the soft palate in these patients, longitudinal studies should be conducted with more participants.

## 7. Conclusion

In conclusion, there was a significant association seen between changes in the shape of the soft palate and the size of the airway. Middle aged patients showed higher UAD, while the older age group showed higher MN-HY. This is one of the first studies done and will be useful in predicting the risk of OSA in different age groups and further long-term studies should be done to better predict the condition.

## Figures and Tables

**Table 1:** Distribution of age groups and shape of soft palate among UAD and MN-HY.

Variables	UAD				MN-HY			
	Mean	SD	Min	Max	Mean	SD	Min	Max
Age groups								
10–30 years	12.07	4.18	3.36	28.72	10.52	4.71	0	24.56
31–50 years	12.54	3.74	5.9	28.72	12.62	5.65	3.72	24.56
Above 50 years	11.42	2.99	5.85	20.88	13.23	6.22	2.91	24.56
Shape of soft palate								
Rat tail shaped	11.45	2.91	5.9	18.93	11.62	4.95	0	23.38
Crocked appearance	12.9	2.68	7.86	19.59	10.85	3.94	1.54	19.25
Butt-like shaped	11.47	3.68	3.36	16.74	7.35	4.75	2.01	20.59
Leaf shaped	10.96	3.68	5.37	20.88	13.87	5.8	1.64	24.56
S-shaped	19.63	8.14	9.16	28.72	9.64	3.25	3.55	20.31
Straight line	12.12	3.56	7.18	20.88	13.94	5.8	4.64	22.3
Undefined	8.54	1.66	7.71	11.03	8.32	3.45	6.6	13.5

Abbreviations: Max, maximum; Min, minimum; SD, standard deviation.

**Table 2:** Association of shape of soft palate with age group.

Shape of soft palate		Age groups			*χ* ^2^ value	*p*-Value
		10–30 years	31–50 years	Above 50 years		
Butt-like	*N*	77	23	8	84.52	<**0.001*⁣*^*∗*^**
	%	71.30	21.30	7.40		
Crooked type	*N*	51	34	4		
	%	57.30	38.20	4.50		
Leaf shaped	*N*	120	25	4		
	%	80.50	16.80	2.70		
Rat tail shaped	*N*	299	62	32		
	%	76.10	15.80	8.10		
S-shaped	*N*	25	16	3		
	%	56.80	36.40	6.80		
Straight line	*N*	17	12	15		
	%	38.60	27.30	34.10		
Undefined	*N*	4	0	0		
	%	100.00	0.00	0.00		

*Note: N*, number of samples. The bold values and asterisk (*⁣*^*∗*^), indicates that the result is statistically highly significant. *χ*^2^ value, Chi-square value.

**Table 3:** Multivariate linear regression with UAD and MN-HY as the dependent variables and age groups and shape of soft palate as the independent variable.

Hypothesis	Regression weights	*R* ^2^	*B*	Std. error	Beta coefficient	*p*-Value
H_1_ 1	Age groups → UAD	0.001	−0.057	0.223	−0.009	0.797
H_1_ 2	Age groups → AN-HY	0.038	1.607	0.28	0.195	**<0.001*⁣*^*∗*^**

*Note:* H_1_ 1: There is a significant impact of age groups on UAD. H_1_ 2: There is a significant impact of age groups on MN-HY beta means standardized partial regression coefficient. Age groups: 10–30 years = 1; 31–50 years = 2; above 50 years = 3. H_1_, alternate hypothesis; AN-HY, mandibular plane hyoid bone. The bold values and asterisk (*⁣*^*∗*^), indicates that the result is statistically highly significant.

Abbreviation: UAD, upper airway diameter.

**Table 4:** Association of shape of soft palate with UAD and MN-HY range.

Airway measurement		Shape of soft palate							*χ* ^2^ value	*p*-Value
		Undefined	Leaf shaped	Rat tail	Butt-like	Straight line	S-shaped	Crooked appearance		
UAD										
Abnormal range of UAD	*N*	4	116	343	85	33	38	78	13.961	**0.03*⁣*^*∗*^**
	%	100.00%	77.90%	87.30%	78.70%	75.00%	86.40%	87.60%		
Normal range of UAD	*N*	0	33	50	23	11	6	11		
	%	0.00%	22.10%	12.70%	21.30%	25.00%	13.60%	12.40%		
MN-HY										
Abnormal range of MN-HY	*N*	4	149	381	108	34	44	83	64.504	**<0.001*⁣*^*∗*^**
	%	100.00%	100.00%	96.90%	100.00%	77.30%	100.00%	93.30%		
Normal range of MN-HY	*N*	0	0	12	0	10	0	6		
	%	0.00%	0.00%	3.10%	0.00%	22.70%	0.00%	6.70%		

*Note: N*, number of samples. The bold values and asterisk (*⁣*^*∗*^), indicates that the result is statistically highly significant. *χ*^2^ value, Chi-square value.

## Data Availability

The data used to support the findings of this study are available from the corresponding author upon request.
